# Nonrandom Missingness in Child Race and Ethnicity Records and the US Federal Data Standards: Pooled Analysis of Community-Based Child Health Studies

**DOI:** 10.2196/65660

**Published:** 2025-11-13

**Authors:** Danielle M Krobath, Norbert L W Wilson, Adolfo G Cuevas, Elena N Naumova, Jennifer M Sacheck, Sydney A Tyler, Christina D Economos

**Affiliations:** 1 Department of Epidemiology and Biostatistics, Arnold School of Public Health, University of South Carolina, 915 Greene Street, Columbia, SC, 29208, United States, 1 8037777353; 2 Friedman School of Nutrition Science and Policy, Tufts University, Boston, MA, United States; 3 Duke Divinity School and Sanford School of Public Policy, Duke University, Durham, NC, United States; 4 Department of Social and Behavioral Sciences, School of Global Public Health, New York University, New York, NY, United States; 5 Center for Anti-racism, Social Justice, and Public Health, New York University, New York, NY, United States; 6 Department of Community Health, Tufts University, Medford, MA, United States; 7 Department of Social and Behavioral Sciences, School of Public Health, Brown University, Providence, RI, United States; 8 Department of Public Health and Community Medicine, School of Medicine, Tufts University, Boston, MA, United States

**Keywords:** race and ethnicity, health disparities, population health, health surveillance, child health, racism, survey research methods, demography, data harmonization, data crosswalk

## Abstract

**Background:**

Racism perpetuates the unequal distribution of power, resources, and privilege within and between societies to the detriment of marginalized groups. Racialization involves categorizing people based on traits to which socially constructed meaning and value have been ascribed. In public health, this process can manifest when tracking racial health disparities in children, which requires aggregating parent-reported race and ethnicity data into federally recognized categories. The demographic surveys used to characterize children’s identity in the United States mirror those administered in adults and typically follow federal race and ethnicity data standards, which include ambiguous response options (eg, other race), “select all that apply” directives, and open-ended fields followed by a request specification, with limited guidance for coding and interpretation. These methodological challenges could contribute to nonrandom data missingness and misclassification bias and must be resolved to better harmonize historic data, especially given recent revisions to the country’s federal race and ethnicity data standards.

**Objective:**

We aimed to explore the prevalence of systematic bias within past, current, and recently revised federal race and ethnicity data standards in the United States and develop a standardized method for improving the reporting of child race and ethnicity in public health research, policy, and practice.

**Methods:**

We developed a replicable decision-making process to uncover racial heterogeneity obscured by key components of US federal race and ethnicity data standards (open-ended and ambiguous response fields). We applied it to a pooled sample of 8 community-based child health studies with 8087 participants and examined changes in the dataset’s racial and ethnic diversity.

**Results:**

Overall, 93.11% (7530/8087) of parents provided child race and ethnicity data, with 3.73% (281/7530) identified as other race and 9.72% (732/7530) identified as multiracial. In total, 101 distinct open-ended written responses (eg, “Haitian”) were provided. The replicable decision-making process resulted in 4.02% (303/7530) of children being reallocated from their parent-reported race or ethnicity category, of whom 38.6% (117/303) were moved into the Black category based on written responses. Within the multiracial group, we identified 22 unique combinations, including White-Hispanic (269/732, 36.7%) and White-Black (169/732, 23.08%).

**Conclusions:**

These findings demonstrate how the current paradigm of assessing race and ethnicity in the United States may contribute to the erasure and further marginalization of individuals disproportionately enduring the effects of racism. While updated federal race and ethnicity data standards may soon take effect, persistent gaps in demographic and health surveillance will remain. Our data reallocation decision-making process offers a novel and practical framework for harmonizing race and ethnicity data across time, populations, and datasets, emphasizing the relevance and longevity of preexisting datasets and tools. Efforts to build equitable public health surveillance and data systems should expand the survey response options, avoid aggregating diverse populations, and develop new statistical techniques for data analysis.

## Introduction

### Conceptualizing Race, Ethnicity, and Health Inequities

Race and ethnicity are social constructs associated with health inequities through the immediate and intergenerational effects of racism, discrimination, and other social hierarchies. Multiple forms of racism permeate society and impede the health of racially minoritized populations, including through interpersonal discrimination, segregated schools and neighborhoods, and discriminatory health care systems [1-6]. These long-standing injustices received renewed global attention in response to the COVID-19 pandemic and ongoing police killings of Black individuals in the United States. Many major institutions (governments, funders, journals, and universities) have also pledged to enact change [7,8]. For instance, the United Nations recently called upon countries to use a “whole-of-government” approach to dismantle structural racism, beginning with the population-level collection, surveillance, and public reporting of racial and ethnic identity data [9].

### Global Approaches to Race and Ethnicity Data Collection and Implications for Health Equity

According to Morning [10], who analyzed unpublished data compiled by the United Nations, only 63% of the 141 countries that fielded national census surveys asked about ethnicity, race, or a related identity construct. However, these terms were differently conceptualized and highly conflated. Most national censuses used the term “ethnicity,” but “race” and “nationality” were often either excluded or measured with identical response categories. Limited quality or nonexistent racial and ethnic data infrastructure allows systemic racism to manifest, driving racial health inequities in the United States and elsewhere around the world [11,12].

Racialization is the process by which individuals are ascribed to a racial or ethnic group based on both self-identified and socially perceived (ie, the categorization of an individual by members of a society) categorizations. Race and ethnicity are complex and dynamic constructs that change by time, place, and historical context [4,13]. For example, Morning [10] found that 13 nations included a census question assessing “race” alone, 11 of which were New World former slaveholding nations (ie, the United States) or their territories [14]. Conversely, many governments—predominantly in Western Europe and West Africa—prohibit official racial or ethnic categorization by the government due to violent histories built upon racial caste systems. The tangible consequences of racialization on health disparities are numerous. Some of the social and economic ramifications in the United States include unequal resource allocation (eg, health care providers may be less likely to accept Medicaid in low-income, racially segregated neighborhoods where the majority of residents are non-White) [15] as well as disproportionately heightened exposure to stress among Black individuals with darker skin tones compared to their White and lighter-skinned counterparts, which can increase allostatic load (ie, the cumulative “wear and tear” on the body resulting from prolonged stress), [16].

Categorizing people by race is crucial for cultivating representative study samples, developing inclusive and effective policies and programs, and alleviating racial health disparities [17-19]. Williams and Jackson [20] have long substantiated that making progress toward racial equity requires collecting and reporting on racial identity. Given that funders either require or encourage collecting data disaggregated by race and ethnicity and medical journals have growing attention on the subject of racism [4,21], researchers and policy makers across the globe may begin or continue reporting race and ethnicity in the context of health outcomes and disease prevalence [21,22].

### Race and Ethnicity Data Systems in the United States

The US government has a well-established demographic surveillance system that simultaneously guides national race and ethnicity data standards. The entities and individuals worldwide that begin collecting race and ethnicity data may turn to investigators experienced in demographic enumeration for methodological insights. While this decision should be made cautiously, the federal data systems in the United States may serve as a starting point for surveilling racial and ethnic health outcomes.

In the United States, the Office of Management and Budget (OMB) Directive 15, *Race and Ethnic Standards for Federal Statistics and Administrative Reporting*, defines the data standards and minimum categories for reporting race and ethnicity for all federal agencies and federally funded health researchers. In 2024, the OMB announced several updates aimed at improving question format, wording, and inclusivity of federal race and ethnicity data standards which are expected to take effect with the 2030 census [23]. Changes will include the creation of a new racial group specifically for Middle Eastern and North African (MENA) individuals and assessing race and Hispanic or Latino ethnicity with a single combined question. However, enumeration of race and ethnicity will still rely on nondescript collective terminology, such as “other race,” and instructing respondents to “select all that apply” to assess and subsequently aggregate diverse individuals into a single residual multiracial category. The multiracial population in the US census increased by 276% over the last decade [24]. The National Institutes of Health acknowledges that individuals of two or more races have unique health risks in the United States, yet standard guidance in data reporting is nonexistent beyond aggregation into a finite category [25]. The OMB standards will continue to allow for open-ended written responses to capture additional information; however, standard guidance for handling subsequent information is nonexistent.

### Limitations of Current Race and Ethnicity Data Collection and Reporting Procedures

While federally-funded health researchers in the United States are required to collect demographic data from study participants, it appears that most peer-reviewed studies published in major epidemiology and public health journals in recent years have not actually used these variables as they fail to report the racial and ethnic composition of study samples or clarify how these constructs were defined and operationalized in practice [26,27]. In pediatric health research, the racial identities of children are usually reported by parents in adherence with the standard OMB Directive 15 guidelines. Rees et al [28] found that most youth identified by their parents as other race were either excluded entirely from analyses or aggregated inconsistently across pediatric health studies. In the 2020 census, the other race was the fastest-growing racial group in the United States: 49.9 million Americans identified as other race either alone or in combination with another racial category [29,30]. Among those classified as other race, 90.8% of individuals were Hispanic or Latino [30]. Hispanic and Latino individuals experience significant health disparities, including the onset of hypertension, obesity, anxiety, and posttraumatic stress disorders at rates that outpace their non-Hispanic White counterparts. The risk for developing these poor health outcomes is even more pronounced among undocumented populations [31]. Even less is known about the implications of aggregating multiracial individuals into a single combined category because very few previous studies in either the pediatric or general health literature have attempted to systematically examine the heterogeneity hidden within this population. As a result, the interrelationship between survey response formats, nonrandom race and ethnicity data missingness, and health outcomes across and within racialized groups remains poorly understood, hindering the potential for public health systems to equitably protect population health.

Overall, the absence of, opposition to, and significant differences between racial and ethnic data collection systems across the globe leave racialized health inequities unrecognized and overlooked. Greater attention by the public health community to race and ethnicity data standards could contribute toward eliminating preventable racial health inequities by accurately identifying and tracking health outcomes to inform inclusive and equitable programs, policies, and interventions [17,32,32,33]. Therefore, this study developed a standardized and replicable method for improving the use and specificity of child race and ethnicity data collected from parent-reported demographic surveys. We demonstrate the implications of investigating the hidden diversity within the open-ended written response fields, other race group, and aggregate multiracial category on demographic surveys. We hypothesized that these oft-applied survey procedures systematically fail to identify specific marginalized populations that are disproportionately exposed to racism. To test our hypotheses, we retrospectively pooled the demographic data of children we enrolled in health and nutrition studies across the United States and examined whether common survey procedures were associated with the misrepresentation of minoritized racial and ethnic groups.

## Methods

### Study Design and Data Source

This cross-sectional study used data from 8087 parents or guardians who reported child demographics at or before baseline in 8 pediatric health studies led by members of our study team. The studies used a community-based design in 6 states across the United States (California, Kentucky, Massachusetts, Mississippi, South Carolina, and Rhode Island) from 1999‐2018. The goal of each study was to improve children’s nutritional health outcomes or health behaviors. Additional details on the individual studies in this pooled analysis are in Table 1.

**Table 1.** Descriptions of the 8 studies included in the pooled analysis of parent-reported race and ethnicity of child participants (N=8087).

Study^a^	Years	State(s)^b^	Setting	Target population	Consented participants, n (%)	Study design	Study outcome(s)
A	2016‐2018	MA	Urban and suburban	Children in grades 3‐4	983 (12.1)	Randomized control trial	Impact of 2 physical activity programs on school time and total daily moderate-to-vigorous physical activity
B	2013	MA	Urban	Children in grades 3‐6	448 (5.5)	Observational cross-section study	Associations between physical activity and academic achievement
C	2011‐2012	MA	Urban	Children in grades 4‐8	693 (8.6)	Randomized control trial	Impact of vitamin D supplementation on serum 25(OH)D and cardiovascular disease risk factors
D	2009‐2010	MA	Urban	Children in grades 4‐8	560 (6.9)	Observational cross-section study	Differences in physical fitness levels and health status
E	2008‐2009	CA, KY, MS, and SC	Rural	Children in grades 1‐6	1301 (16.1)	Randomized control trial	Community environmental change intervention on undesirable weight gain
F	2007‐2008	CA, KY, MS, and SC	Rural	Children in grades 1‐6	959 (11.8)	Prepost program evaluation	Changes in nutrition behaviors following an after-school health program implementation
G	2003‐2004	MA	Urban and suburban	Children in grades 1‐3	1726 (21.3)	Community-level intervention with matched control communities	Influence of environmental change intervention on preventing excess weight gain
H	1999‐2002	MA and RI	Urban and suburban	Children aged 6-9 years	1417 (17.5)	Group randomized trial	Impact of a nutrition and physical activity intervention on bone quality and muscular strength

a Study names have been deidentified to protect participant privacy and unpublished data.

b MA: Massachusetts; CA: California; KY: Kentucky; MS: Mississippi; SC: South Carolina; RI: Rhode Island.

### Ethical Considerations

The institutional review board of Tufts University exempted this study from review because it was deemed not human participant research. Study data were deidentified before being shared for pooled analysis, and no direct identifiers (names and contact information) were included. For the 8 pediatric research studies described herein, we obtained informed consent from the parents or caregivers and verbal assent from the youth participants. Across each of the 8 studies, participants received stipends or compensation that varied slightly by protocol (cash, gift cards, or equivalent stipends). All compensation procedures were approved by the Tufts University institutional review board. We followed the Strengthening the Reporting of Observational Studies in Epidemiology (STROBE) reporting guidelines (Checklist 1).

### Harmonizing Data Across Diverse Race and Ethnicity Survey Response Formats

**Figure 1.**
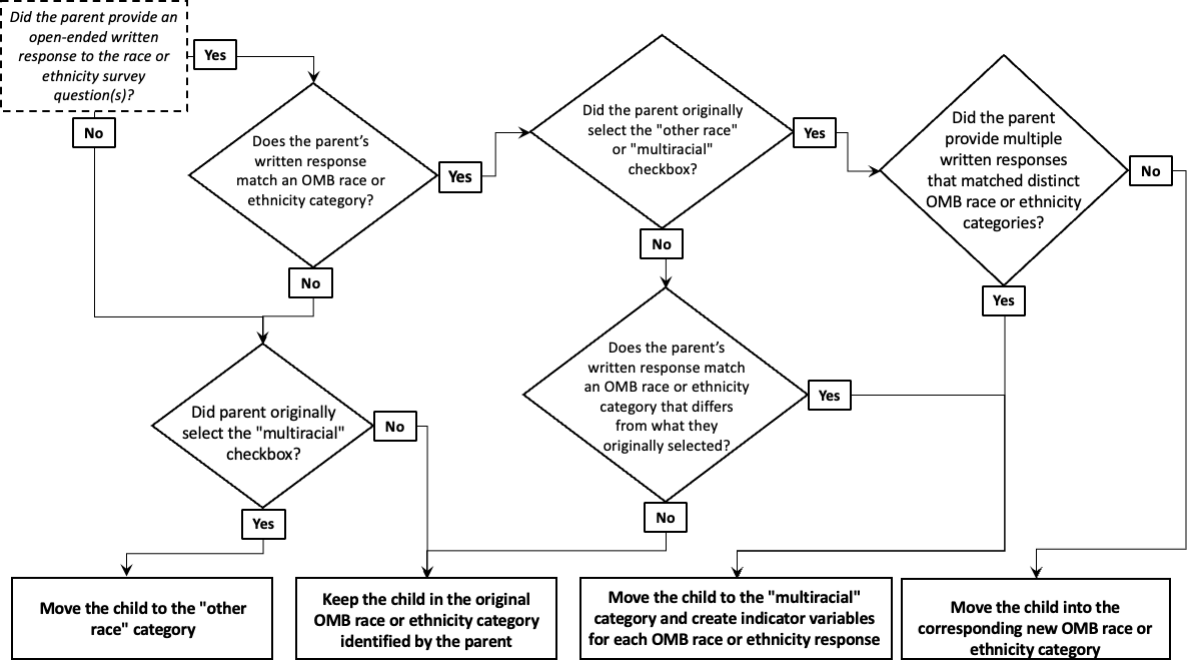
Decision-making process used to allocate all parent-provided written and categorical responses to child race and ethnicity to the Office of Management and Budget (OMB) Directive 15 standards. This flowchart illustrates the standardized procedure we created to ensure consistent categorization of race and ethnicity data across 8 community-based studies with varying race and ethnicity data collection instruments. The decision-making process represents a replicable coding procedure for mapping parent or caregiver-reported child race and ethnicity responses to the federal OMB Directive 15 standards. The process accounts for single- and multiple-race responses, open and close-ended responses, use of the other race and multiracial checkboxes, and written responses that match or diverge from existing OMB categories.

Inevitably, surveys that ask diverse individuals with unique life experiences to self-report their racial and ethnic identities in limited detail to facilitate group comparisons flatten the complex identities of respondents [10,34,35]. However, our overall objective is to illustrate how the current paradigm of collecting, reporting, and operationalizing the social constructs of race and ethnicity in the United States can bias demographic estimates of populations and may produce inaccurate research findings, which ultimately obscures understanding of the prevalence of racial health disparities. To harmonize children’s race and ethnicity data, we pooled and assessed parent-reported survey responses in a double-blinded process (researchers were unaware of the categorical responses when allocating written responses). Figure 1 depicts our replicable decision-making process.

The individual race and ethnicity survey response formats replicated or exceeded the federal minimum reporting categories in OMB Directive 15 [26]. Details on survey response options are in Table 2. As observed in surveys globally [10], all demographic surveys offered a set of closed-ended race and ethnicity categories and the other race checkbox option [36]. The closed-ended race and ethnicity categories included White (White or Caucasian), Black or African American, Asian or Pacific Islander (Asian, Asian American, Asian Indian, or Native Hawaiian or Other Pacific Islander [NHPI]), American Indian or Alaska Native (Native American, American Indian, or Alaska Native), and Hispanic or Latino (Hispanic, Latino, Spanish, or Mexican). All surveys accepted open-ended written responses designated with “please specify.” We allocated individuals to the multiracial category if parents or guardians (1) selected the multiracial checkbox (as was the status quo for studies conducted in early years), (2) selected multiple categories, or (3) selected a single category and wrote a distinct race or ethnicity in the “please specify” field (Table 2).

**Table 2.** Examples of open-ended responses in “please specify” fields and how they were allocated according to federal US race and ethnicity standards.^a^^,^^b^

Example written responses	Official race or ethnicity category definition in the federal register
Any federally recognized tribe (ie, “Cherokee”)	American Indian or Alaska Native: A person having origins in any of the original peoples of North and South America (including Central America) and who maintains tribal affiliation or community attachment.
Indian, Japanese, or Vietnamese	Asian: A person having origins in any of the original peoples of the Far East, Southeast Asia, or the Indian subcontinent, including, for example, Cambodia, China, India, Japan, Korea, Malaysia, Pakistan, the Philippine Islands, Thailand, and Vietnam.
Haitian, Malian, or West African	Black or African American: A person having origins in any of the Black racial groups of Africa. Terms such as “Haitian” or “Negro” can be used in addition to “Black or African American.”
Central American, El Salvadorean, or Puerto Rican	Hispanic or Latino: A person of Cuban, Mexican, Puerto Rican, Cuban, South or Central American, or other Spanish culture or origin, regardless of race. The term, ‘‘Spanish origin,’’ can be used in addition to ‘‘Hispanic or Latino.’’
Polynesian, Hawaiian, or Tahitian	Native Hawaiian or Other Pacific Islander: A person having origins in any of the original peoples of Hawaii, Guam, Samoa, or other Pacific Islands.
Austrian, Dutch, or Lebanese	White: A person having origins in any of the original peoples of Europe, the Middle East, or North Africa.

a The table provides example written responses that parents provided regarding their child’s race and ethnicity, along with the corresponding federal race or ethnicity category into which the answer was categorized.

b Example written responses that do not map to federal race or ethnicity categories: American, Brazilian, Jewish, Muslim, the United States, Massachusetts, and mixed.

In 6 of the 8 studies, demographic surveys assessed race and ethnicity (Hispanic or Latino, not Hispanic or Latino) using a single combined question, reflecting best practices during the respective year(s) of implementation. In studies A and B (1431/7530; 17.68% of the pooled dataset), which were the only ones that assessed race and ethnicity separately, 51.40% (735/1431) of children were identified as Hispanic or Latino. However, racial identity data were missing for 66.8% (491/735) of those identified as Hispanic or Latino in these studies A (the question was left blank or the other race box was selected). To ensure consistency across studies, we grouped ethnicity and race responses such that multiracial includes children identified as two or more federally recognized races, including Hispanic or Latino.

### Using Open-Ended Responses to Improve the Accuracy of Race and Ethnicity Data

Next, we individually examined and harmonized written and categorical race or ethnicity responses in a similarly double-blinded fashion to understand the groups misrepresented by response formats. We attempted to allocate the open-ended written responses according to federal race and ethnicity categories, modeled after similar approaches by the US Census Bureau [37-39]. The research team discussed discrepancies and resolved them through consensus, guided by the OMB Directive 15 standards [40]. Our use of the term “[re]allocation” in the context of this study is not meant to discount the parent or guardian’s original response. Rather, we recognize that the open-ended responses from respondents reflect essential aspects of children’s identity that warrant consideration by researchers.

### Disaggregating Individual Race or Ethnicity Responses Within the Aggregate Multiracial Group

To demonstrate the breadth and depth of information lost by grouping diverse individuals in a single multiracial category, we disaggregated and plotted the individual responses within the multiracial group using an UpSet plot. This approach visually highlights both the diversity and the structure of the multiracial category.

### Statistical Analysis

Individuals were the primary unit of analysis. We examined changes in racial and ethnic group frequencies (as defined by the OMB 15 Standards) before and after applying our standardized allocation process described in Figure 1. Chi-square tests determined whether there was a significant association between the original parent-reported race or ethnicity category and the reallocated category. Analyses were conducted from June 1, 2022, to July 26, 2023, using Stata/SE software (version 17; StataCorp LLC).

## Results

### Analytic Sample Characteristics

**Figure 2.**
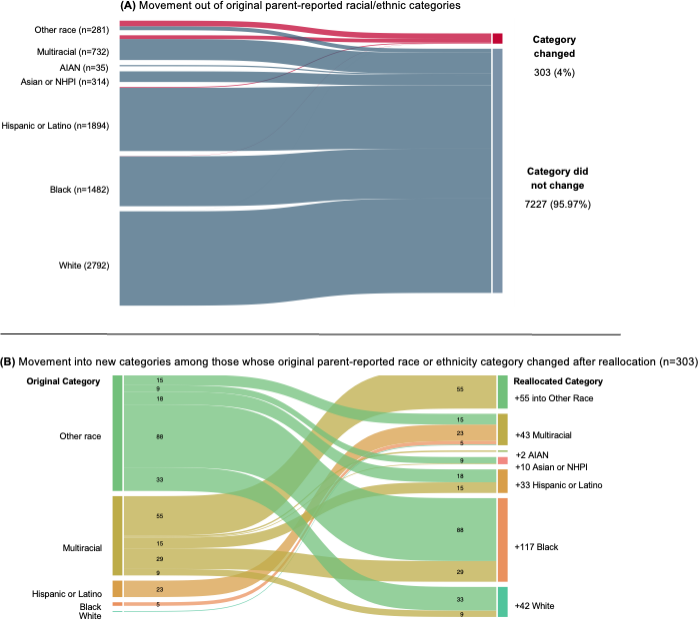
Results of reallocation process on frequency of and movement between racial and ethnic groups. (A) Movement out of original parent-reported racial or ethnic categories. (B) Movement into new categories among those whose original parent-reported race or ethnicity category changed after reallocation (n=303). AIAN: American Indian or Alaska Native; NHPI: Native Hawaiian or Other Pacific Islander.

In total, 93.11% (7530/8087) of parents provided data on the child’s race or ethnicity, and 6.88% (557/8087) did not (fields were left blank or demographic surveys were not returned). Altogether, the open-ended written responses to race or ethnicity spanned 101 distinct groups, including responses such as “Haitian,” “Mexican,” and others (Table 2). Racial and ethnic characteristics of the sample before and after the harmonized reallocation process are in Table 3. Implementing this process (Figure 1) revealed that 95.97% (7277/7530) of children remained in their original parent-reported race or ethnicity category; the chi-square test indicated a strong and significant association between the original and reallocated racial categories (*χ*²_36_=37,000; *P*<.001). Figure 2 shows that of the 4.02% (303/7530) of participants who were reallocated, 89.4% (271/303) were originally identified as multiracial or other race by their parents. Figure 2 also illustrates the movement across racial groups among the subsample of participants who were reallocated out of the original parent-reported category.

**Table 3.** Cross-tabulation of original parent-reported child race or ethnicity with child race or ethnicity after the replicable reallocation process^a^.

	Reallocated racial or ethnic group, n							
	White	Black	Hispanic	Asian or NHPI^b^	AIAN^c^	Multiracial	Other race	Total
Original parent-provided categorical response for the race or ethnicity of the child, n								
White	2791	0	0	0	0	1	0	2792
Black	0	1477	0	0	0	5	0	1482
Hispanic	0	0	1871	0	0	23	0	1894
Asian or NHPI	0	0	0	314	0	0	0	314
AIAN	0	0	0	0	35	0	0	35
Multiracial	9	29	15	1	2	621	55	732
Other race	33	88	18	9	0	15	118	281
Total	2833	1594	1904	324	37	665	173	7530

a *χ*²_36_=37,000; *P*<.001.

b NHPI: Native Hawaiian or Other Pacific Islander.

c AIAN: American Indian or Alaska Native.

### Reallocation Results From the Harmonized Reallocation Process

Among youth originally categorized as other race, only 41.9% (118/281) remained in this category after harmonization, while 31.3% (88/281) were moved into Black, 11.7% (33/281) into White, 6.4% (18/281) into Hispanic or Latino, 5.3% (15/281) into multiracial, and 3.2% (9/281) into Asian or NHPI. Moreover, 84.8% (621/732) of children who were originally identified as multiracial remained in the same group after the harmonization process, while 3.9% (29/732) were reallocated to Black, 2% (15/732) to Hispanic or Latino, and 7.5% (55/732) to other race.

Following the reallocation process, the Black category experienced the largest increase, with 117 additional children reclassified into this group, increasing from 1482 to 1594 (a +7.42% change). This change reflected the movement of 88 children originally categorized as other race and 29 as multiracial into the Black category, while 5 children originally identified as Black were reallocated to the multiracial group. Alternatively, the other race category experienced the largest decrease, going from 281 to 173 (a –38.43% change). This change reflected that 55 children originally identified as multiracial were moved into the other race category because their parents selected the multiracial checkbox without providing additional written details to create indicator variables. However, 163 children were moved out of the other race group into a different OMB category (15 into multiracial, 9 into Asian or NHPI, 18 into Hispanic or Latino, 88 into Black, and 33 into White). Moreover, the multiracial category declined from 732 to 665 children (a –9.5% change) following the reallocation process for several reasons. As previously described, 109 children left the category (9 moved into White, 29 into Black, 15 into Hispanic or Latino, 1 into Asian or NHPI, and 55 into other race). In addition, 15 children moved into the multiracial category from other race because the parent checked the other race box but provided two or more written responses that were mapped to distinct OMB racial groups, such as French (White) and Japanese (Asian). Another 23 children were reallocated into the multiracial category from Hispanic or Latino because the parent provided an open-ended written response that mapped to a distinct OMB category and either checked the Hispanic box or answered affirmatively to the question of whether the child was Hispanic or Latino (a child with a check in the Hispanic box with “White and Black” written would be allocated to multiracial with indicators for Hispanic or Latino, White, and Black). In addition, 5 children moved from Black to multiracial because the parent checked Black and provided a written response that would be defined as a distinct category per the OMB standards. The White category increased by 41 children, from 2792 to 2833 (a +1.47% change), due to 33 children joining this category who were originally identified as other race (parent could have checked the other race box and wrote “German”) and 9 as multiracial (in studies prior to 2000, the parent checked multiracial and could have written “German”), while 1 was moved from White into multiracial. The Asian or NHPI category increased by 10 children, rising from 314 to 324 (a 3.2% increase), largely due to the 9 children who were reallocated to this group from other race.

### Disaggregating the Racial or Ethnic Combinations Within the Multiracial Category

**Figure 3.**
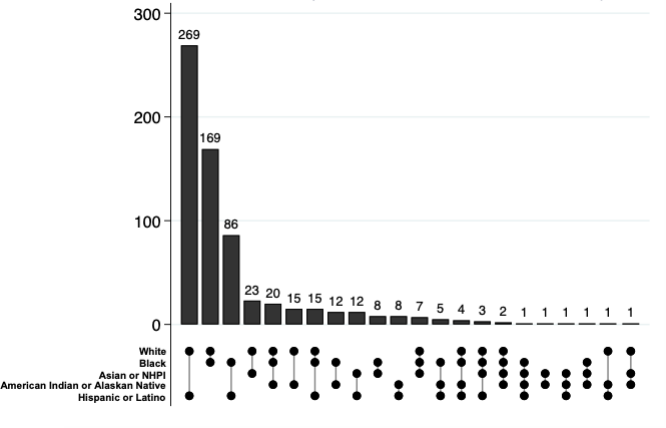
Prevalence and overlap of racial or ethnic identity combinations within the multiracial group (n=665). The vertical bars represent the number of participants identified with each specific combination of racial and ethnic identities, with bar height indicating the number of participants within that combination. The horizontal matrix below the bars shows which identities are included in each of the 22 distinct combinations. NHPI: Native Hawaiian or Other Pacific Islander.

Figure 3 presents an UpSet plot illustrating the overlap among the 22 distinct racial and ethnic identity combinations we uncovered within the 8.83% of participants (665/7530) categorized as multiracial in the reallocated dataset (N=8087). For example, the bar with markers for White, Black, and Hispanic or Latino represents multiracial participants who were reported with all 3 identities. Among the multiracial participants, most were White and Hispanic or Latino (269/665, 40.5%). The next most common combination was White and Black (169/665, 25.4%) individuals, followed by Black and Hispanic or Latino (86/665, 12.9%). The American Indian or Alaska Native identity contributed to 13 of the 22 combination categories, but only 12.2% (81/665) of multiracial children held this identity.

## Discussion

### Principal Findings

Retrospectively harmonizing survey data across 8 community-based child health studies revealed new insights into how past, current, and new federal data standards can influence the representation of marginalized racial and ethnic populations in public health research, policy, and practice. We observed significant changes in the racial and ethnic composition of our sample after reallocating parent-provided written and categorical responses on child race or ethnicity surveys into federally defined categories. Our findings add to the existing literature underscoring how specific components of survey research design disproportionately threaten the quality, completeness, and internal validity of social determinants of health data for minoritized racial populations [41]. The inconsistencies in survey design and methodology we identified may reinforce racial health disparities at the population level through racial group misclassification, nonresponse bias, and the obscuring of diversity through the aggregate multiracial category, thereby warranting further hypothesis testing [42]. Continuing to overlook these survey research elements—open-ended responses, the other race category, and multiracial populations—may hinder racial equity by contributing to an evidence base that lacks validity and reliability for racial and ethnic groups that are exposed to multiple forms of discrimination. The trends we observed between written and categorical responses underscore that race and ethnicity are context-specific, fluid, and dynamic social constructs, which researchers and policymakers should carefully consider during data collection, analysis, and dissemination [5].

Currently, public health researchers and surveillance methods may not adequately account for the confusion that can result from inconsistent or conflated terminology, government standards, and societal norms around race and ethnicity. In addition, internalized racism may lead many immigrants and members of marginalized racial groups to reject or not identify with federal racial or ethnic categories. For example, all surveys in our study enumerated the single combined Black or African American categorical response option; however, many children were reallocated into this category because their parents checked other race and provided written data, such as “Haitian,” “Cape Verdean,” or “Black.” Documented differences in group consciousness between foreign and US-born Black individuals could partially explain this discrepancy [43]. Intraracial relations also play a prominent role in how Caribbean immigrants, African immigrants, and US-born Black people view themselves and each other, underscoring that heterogeneity within racial, ethnic, and nativity groups shapes individual identity formation [44-47]. Cultural demands and expectations profoundly affect identity formation, particularly in how an individual chooses to identify with a larger demographic group [46,48-50]. The findings of this study may also inform broader efforts to assess data related to descendants of formerly enslaved individuals in the United States, which was a major point of contention during the OMB’s process of updating the race and ethnicity data standards announced in 2024. While this was identified as a priority area for further research, the final decision was not to require the collection of such data given concerns that doing so could make accurately counting the Black community, and particularly African immigrants, even more difficult [23]. Overall, our results raise important directions for new research examining how racism, and specifically anti-Blackness and settler colonialism, impact demographic surveillance systems and racial health research in the United States and elsewhere, across generations and age groups [43].

Our findings also underscore the need to improve the measurement and reporting of child race and ethnicity. Following the current best practice in pediatric health research, all surveys asked parents or caregivers to report the racial or ethnic identity of their child. However, racial identity formation is influenced by societal norms and intrafamilial characteristics, such as maternal race and acculturation levels. Therefore, many children, especially those from immigrant, Black, and Hispanic families, might have self-identified their race differently than did their parents if they were administered similar demographic surveys. There is a major gap in knowledge regarding the prevalence and implications of discrepancies between how parents and children identify themselves and each other. Therefore, the understanding of demographic trends among youth and the ability to address racial health disparities could be improved by collecting the self-identified and parent-identified race and ethnicity of children, and recollecting this data over time to account for changes during critical periods of social identity development [51-53].

Our results also reinforce the broader movement by MENA individuals in the United States for official federal recognition with their own ethnicity category [54]. We allocated 33 participants from other race to White due to written responses indicating MENA identity (eg, “Lebanese”). People of MENA descent have diverse backgrounds, cultures, and experiences [54]. MENA individuals in the United States and other Western countries do not identify as White, are often not socially perceived as White, and endure pervasive yet specific forms of discrimination and xenophobia, and the federal government’s categorization of them as “White” is inappropriate [54]. Aggregation of heterogeneous individuals into broad racial or ethnic groups mutes the diversity of populations. Some states have enacted laws to prevent the harms of aggregation for some but not all minoritized racial groups. For example, the *California Government Code* Section 8310.5 requires all state agencies in California to collect data for each major Asian and Pacific Islander group (ie, including, but not limited to, Asian Indian, Cambodian, Chinese, Filipino, Guamanian, Hawaiian, Hmong, Japanese, Korean, Laotian, Samoan, Tahitian, and Vietnamese) [55]. Detailed subgroup reporting should be mandated nationally, extending beyond Asian subpopulations to include MENA, people racialized as Black, diverse individuals of Hispanic and Latino heritage, and others. By examining written responses, we substantiated the high probability of widespread nonrandom missingness of race or ethnicity data associated with close-ended survey response options. Essentially, these survey techniques erase the existence and experiences of members of minoritized racial groups, representing a ubiquitous form of racialized social hierarchies.

The geographic areas within which our studies worked included large populations of Brazilians. Many individuals checked other race and wrote in “Brazilian” but could not be allocated to a specific racial or ethnic group because the current OMB race and ethnicity standards do not recognize Brazilians. Some Brazilians may self-identify as Latino due to Brazil’s geographic location in South America; however, the OMB definition of Hispanic or Latino, which “refers to a person of Cuban, Mexican, Puerto Rican, South or Central American, or other Spanish culture or origin, regardless of race,” specifically excludes this group [56]. Brazil was colonized by the Portuguese, who the OMB currently defines as White, or “a person having origins in any of the original peoples of Europe, the Middle East, or North Africa,” which overlooks and fails to acknowledge the effects of colonization over history. The complexities of racialization among Brazilians in the United States are far-reaching. For instance, the US Census Bureau explicitly excludes Brazilians from its definition of “Hispanic or Latino” and even back codes their data (ie, recategorizes Brazilians as non-Hispanic or Latino even if they self-identified as Hispanic or Latino) in federal reports [57]. However, the National Institutes of Health style guide explicitly mentions Brazilians when describing the Latino category [58]. Nonetheless, federally recognized racial and ethnic groups are not inclusive toward Brazilians, who are recategorized as other race, non-Hispanic during US Census Bureau data cleaning and processing, effectively rendering them invisible. Further research with Brazilian communities is needed to guide the creation of more inclusive race and ethnicity data standards and to avoid additional obscurement of the unique health risks and protective factors within this population, which rose by nearly 50% in the United States between 2010 and 2019 and is projected to continue growing [59].

Marginalized populations deeply affected by racism are obscured by the aggregate multiracial category. Aggregation involves the loss of information and decreased ability of statistical and epidemiological tools to analyze and identify patterns in the outcome of interest [32]. Frequently, a simplistic aggregation approach of multiracial individuals is used to simplify data collection or, presumably, to protect individual privacy. However, these aggregation decisions accomplish neither in practice nor in theory [60]. The disaggregation approach reported in our study could be replicated at a national scale using existing cohorts to improve our understanding of the experiences of populations historically excluded from health research. Because very little attention has been paid to the heterogeneity within biracial and multiracial populations, the scope and specific implications of the aggregate category’s interference with representation and demographic surveillance remain unknown. Critically, more research is needed to develop clear analytical guidelines to improve the representation of diverse individuals.

Our findings have direct application to the procedural transitions that institutions will be faced with implementing as the OMB’s 2024 revisions to race and ethnicity data standards take effect in 2030. Data crosswalks, such as the translational harmonization process we developed, are essential for accurately comparing temporal changes in (1) population prevalence and demographic shifts and (2) health risks, behaviors, and outcomes across racial or ethnic groups. In our pooled analysis, child race and ethnicity data were measured using methods consistent with how child health and demographic estimates have been reported in the United States for more than 3 decades. The approach presented in our study provides a feasible framework to support large-scale harmonization of child race and ethnicity data across time, populations, and datasets. Adopting this process could help mitigate systematic racial bias in federal data standards and safeguard the validity of child health data across past and future generations. Overall, our study highlights and offers a solution for persistent gaps in race and ethnicity data practices that remain despite forthcoming OMB revisions.

### Generating Future Research Directions

This study pooled and harmonized parent-reported data on children’s racial and ethnic identity. Additional research is needed to determine whether distinct patterns emerge when this process is applied to adult populations or to self-reported data from children. Nonetheless, OMB race and ethnicity standards do not differentiate racial or ethnic group definitions or data handling techniques (collection, cleaning, or reporting) based on the age of the population. Therefore, we posit that standard survey formats—a set of close-ended race or ethnicity categories with other race, a “select all that apply” instruction, and an open-ended “please specify” field—incur significant limitations for accurately enumerating race and ethnicity in public health research and surveillance. As a result of identifying specific mechanisms in data collection and reporting, we present 3 hypotheses for future investigation into the overlooked drivers of structural racism in health research.

First, we hypothesize that when survey questions and response formats change to reduce the stigmatizing, incomplete, inconsistent, or unclear language (including language barriers), respondents are less likely to withhold information or identify as a collective category such as other race. [61] Thus, researchers can reduce bias in the data collection stage. With targeted investments by funders, researchers could improve the inclusion of underrepresented groups at the design and input stages. Toward this hypothesis, investigators could systematically test for interlinkages between close- and open-ended responses using our decision-making process (Figure 1) to uncover how aggregate and constrained race and ethnicity categories hide or exclude specific groups. Input from diverse community members must be prioritized during this investigation and when designing race and ethnicity survey questions to prevent additional stigma and bias and build trust with communities [18,62,63].

Second, we hypothesize that when crude aggregation of individuals of multiple races or ethnicities into residual categories is avoided, the bias associated with aggregation at the data analysis and presentation stages could be substantially reduced. Researchers could substantially reduce race-related bias by making relevant decisions that better capture the diversity of multiracial participants and examine the specificity of treatment effect differences. Reliance on aggregation as the practical method of planning and implementing public health research ignores minoritized groups and blocks paths for further investigation. Testing these hypotheses by the global research community may uncover local and universal mechanisms and pathways of how identity obfuscation and invalid assumptions contribute to structural racism and decrease equitable representation in health surveillance, research, policies, and programs.

Third, we hypothesize that the significant health differences that exist across racial and ethnic groups could be more accurately understood and addressed through the large-scale application of this data harmonization and disaggregation method. This approach would allow researchers and health agencies to reanalyze population cohorts, providing deeper insight into how our understanding of health status may be shaped or obscured by racial aggregation, particularly for individuals identified as other race and multiracial.

### Limitations

Our exploratory process aimed to provide a method to inform improved race and ethnicity data systems to reflect more accurately the identities and backgrounds of participants, but this work presents some limitations. First, we used a convenience sample of data by pooling children’s demographic surveys from studies conducted by investigators at Tufts University in Boston, Massachusetts. Thus, while these studies did recruit children living in 6 US states, our results were not meant to be generalizable to the broader pediatric population in the United States or elsewhere. In addition, our harmonized replication process required changing some parents’ initial categorical race and ethnicity responses to different categories. However, the overall implications of this work could allow researchers and policy makers to address health inequities with a consistent and inclusive approach to data collection and reporting. Specifically, retrospectively applying this method to population datasets will reveal groups that require new unique categories instead of being forced to omit data or identify with the long-standing yet ambiguous other race option. Moreover, children whose parents checked the multiracial checkbox but provided a single written response were reallocated into the corresponding single-race category. A drawback of this decision was not preserving these children’s multiracial identity. We acknowledge that grouping multiracial individuals with their monoracial counterparts is not the appropriate solution since histories, experiences, and self-identities within groups differ [34,64]. Going forward, researchers should avoid using a single checkbox to assess the identities of multiracial individuals and work to develop equitable and reflective measurement and analysis practices. Our goal was to contribute to local, national, and global conversations that address the stigmatizing and exclusionary nature of current race and ethnicity data standards while providing a framework to increase data visibility for investigators and governments who may implement new and existing practices.

### Conclusions

Rectifying the past and present harms of racism begins with ensuring the race and ethnicity data that inform research and policy are reliable, replicable, valid, and inclusive. The current paradigm of assessing these social constructs on surveys in the United States significantly impedes the ability to identify, track, and improve health outcomes across and within diverse racial and ethnic groups. The results of our study could inform future changes to the OMB’s formal process for collecting and reporting race or ethnicity in the United States. Our study underscores that Black subpopulations in the United States are also at a disproportionately high risk of misclassification. The implications of federal racial data standards for people racialized as non-Hispanic Black have been largely excluded from policy discussions and recent OMB data standard revisions. Creating new categories that reflect the overwhelming heterogeneity within the Black population (eg, Afro-Caribbeans) must be central within any forthcoming changes to federal race and ethnicity data standards. Simultaneously, researchers across disciplines must develop new methods to account for growing multiracial populations worldwide.

To address growing racial health inequities, we advocate for the large-scale application of our standardized method to improve understandings of disease disparities and inform more inclusive public health research and policy. Such methodological decisions are essential for understanding the causes and consequences of racism and forming evidence-based guidelines to address inequities across the globe. Minoritized racial groups have long endured mistreatment in science and society, hence the ongoing global reckoning with racism [3,63,65,66]. Targeted investments and collective efforts by entire research teams, funders, journals, institutions, and governments are necessary to find, test, and implement solutions that uncover the unique contributions of multiple forms of racism to health outcomes, which are currently unknown due to nonrandom structural missingness of racial identity data. Race, ethnicity, and other forms of identity data inform health policy, research, and programs, and unless corrected, the problems we have identified will exacerbate long-standing health disparities and leave experiences of racial discrimination undocumented and overlooked.

## Supplementary material

10.2196/65660 Checklist 1 STROBE checklist.

## Abbreviations

MENA: Middle Eastern and North African
NHPI: Native Hawaiian or Other Pacific Islander
OMB: Office of Management and Budget
STROBE: Strengthening the Reporting of Observational Studies in Epidemiology
